# CTR > 0.7 predicts the subgroup of lung adenocarcinomas ≤ 2 cm at risk of poor outcome treated by sublobar resection compared to lobar resection

**DOI:** 10.1186/s40644-024-00717-4

**Published:** 2024-06-17

**Authors:** Weiwei Jing, Qi Li, Mengxi Liu, Yi Zhang, Sifan Chen, Ke Zhang, Dan Li, Min Zhao, Yineng Zheng, Wangjia Li, Yangying Wu, Hongbo Xu, Ziya Zhao, Shaolei Kang, Fajin Lv

**Affiliations:** 1 Department of Radiology, The First Affiliated Hospital of Chongqing Medical University, 1 Youyi Rd, Yuanjiagang, Yuzhong District, Chongqing, 400016 People’s Republic of China; 2 Department of Pathology, National Cancer Center/National Clinical Research Center for Cancer/Cancer Hospital, Chinese Academy of Medical Sciences and Peking Union Medical College, Beijing, 100021 China; 3 Department of Pathology, The First Affiliated Hospital of Chongqing Medical University, Chongqing, People’s Republic of China; 4 Department of Radiology, First Affiliated Hospital of Kunming Medical University, 295 Xichang Rd, Wuhua, Kunming, 650032 China

**Keywords:** Lung adenocarcinoma, 2 cm, CTR, Surgical approach, Prognosis

## Abstract

**Background:**

A standard surgical procedure for patients with small early-stage lung adenocarcinomas remains unknown. Hence, we aim in this study to assess the clinical utility of the consolidation-to-tumor ratio (CTR) when treating patients with small (2 cm) early stage lung cancers.

**Methods:**

A retrospective cohort of 298 sublobar resection and 266 lobar resection recipients for early stage lung adenocarcinoma ≤ 2 cm was assembled from the First Affiliated Hospital of Chongqing Medical University between 2016 and 2019. To compare survival rates among the different groups, Kaplan-Meier curves were calculated, and the log-rank test was used. A multivariate Cox proportional hazard model was constructed utilizing variables that were significant in univariate analysis of survival.

**Results:**

In the study, 564 patients were included, with 298 patients (52.8%) undergoing sublobar resection and 266 patients (47.2%) undergoing lobar resection. Regarding survival results, there was no significant difference in the 5-year overall survival (OS, *P* = 0.674) and 5-year recurrence-free survival (RFS, *P* = 0.253) between the two groups. Cox regression analyses showed that CTR ≥ 0.75(*P* < 0.001), age > 56 years (*P* = 0.007), and sublobar resection(*P* = 0.001) could predict worse survival. After examining survival results based on CTR categorization, we segmented the individuals into three categories: CTR<0.7, 0.7 ≤ CTR<1, and CTR = 1.The lobar resection groups had more favorable clinical outcomes than the sublobar resection groups in both the 0.7 ≤ CTR < 1(RFS: *P* < 0.001, OS: *P* = 0.001) and CTR = 1(RFS: *P* = 0.001, OS: *P* = 0.125). However, for patients with 0 ≤ CTR < 0.7, no difference in either RFS or OS was found between the lobar resection and sublobar resection groups, all of which had no positive events. Patients with a CTR between 0.7 and 1 who underwent lobar resection had similar 5-year RFS and OS rates compared to those with a CTR between 0 and 0.7 who underwent sublobar resection (100% vs. 100%). Nevertheless, a CTR of 1 following lobar resection resulted in notably reduced RFS and OS when compared to a CTR between 0.7 and 1 following lobar resection (*P* = 0.005 and *P* = 0.016, respectively).

**Conclusion:**

Lobar resection is associated with better long-term survival outcomes than sublobar resection for small lung adenocarcinomas ≤ 2 cm and CTR ≥ 0.7.

## Introduction

In both China and globally, lung cancer remains the primary reason for cancer-related deaths [1, 2]. Surgical resection is the primary treatment for early stage lung cancer. The LCSG821 prospective study [3] in 1995 demonstrated that lobar resection had a significantly lower local recurrence rate for T1-stage lung cancer than sublobar resection, but also resulted in a significantly higher survival rate.

Although lobar resection is the established surgical approach for early lung cancer, emerging evidence suggests that sublobar resection is gaining recognition in the clinical management of early lung adenocarcinoma. Studies over the last twenty years have shown that GGNs have a better outlook than SNs according to research [4, 5]. Aherne et al. [6] confirmed that ground-glass opacities on HRCT correspond to the pathological lepidic component, and the solid component corresponds to the pathological invasiveness. A clinical trial conducted by the Japan Clinical Oncology Group (JCOG) 0201 [7] demonstrated that a maximum consolidation diameter/maximum tumor diameter (c/t) ratio of ≤ 0.25 in GGNs can effectively forecast the presence of non-invasive lung adenocarcinoma, thereby establishing it as a defined entity known as non-invasive imaging adenocarcinoma. Following this, the JCOG sequentially developed JCOG0804, JCOG0802, and JCOG1211 to investigate the potential of image features to inform clinical decision-making for early lung adenocarcinoma, based on the proportion of ground-glass components. The results for JCOG0201 [8], JCOG0802, and JCOG1211 [9] have been recently published. The survival outcomes of JCOG1211 and JCOG0201 showed that for nodules measuring less than 3 cm with a CTR < 0.5, sublobar resection had similar survival outcomes to lobar resection. Moreover, the survival outcomes of JCOG0802 showed non-inferiority of sublobar resection compared to lobar resection for patients with peripheral stage IA non-small cell lung cancer with a total tumor size ≤ 2 cm and CTR > 0.5. However, the cut-off value of the CTR in deciding whether to undergo surgery was inconsistent across different studies [10, 11].

Therefore, our objective in this research was to assess the practicality of CTR in treating individuals with small (≤ 2 cm) early stage lung adenocarcinoma.

## Methods

### Patients

A review was conducted on 1091 patients with clinical stage IA adenocarcinoma who underwent lobar or sublobar resection at our facility from 2016 to 2019. Exclusion criteria included patients with inadequate data (51 cases), total tumor size exceeding 2.0 cm (389 cases), presence of other malignant tumors (42 cases), lymph node metastasis (22 cases), administration of adjuvant chemotherapy (12 cases), or lack of information (11 cases). In the end, a total of 564 patients with data were included in the research. Institutional Review Board (IRB No. approval was acquired. The need for written informed consent for this retrospective study was waived by the ethics committee (K2023-223) at the First Affiliated Hospital of Chongqing Medical University. The research was carried out following the ethical guidelines of the institution in relation to human participants and in adherence to the Declaration of Helsinki.

### Radiologic evaluation

**Fig. 1:**
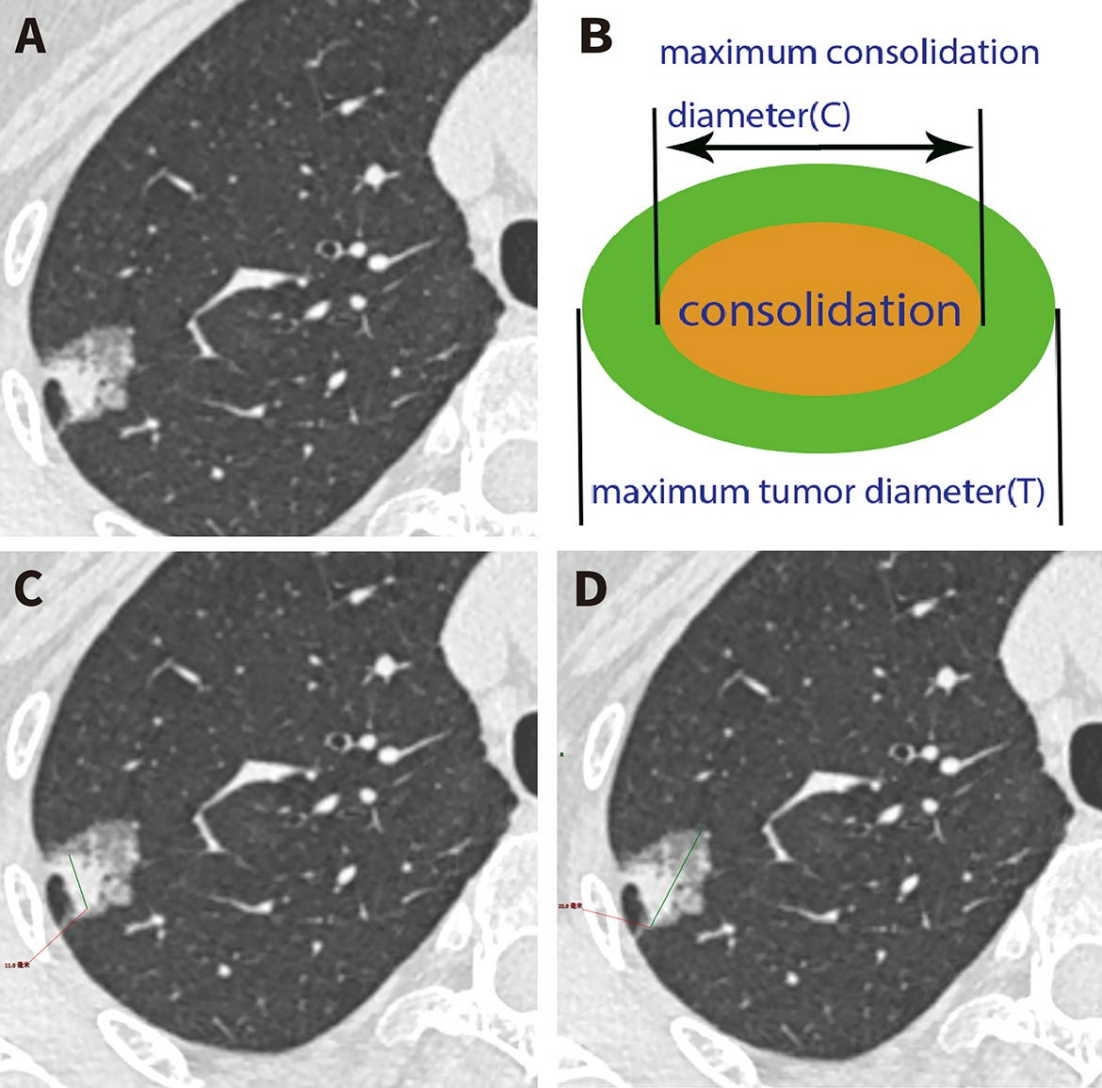
Measurement of the consolidation-to-tumor ratio (CTR). **A** Computed tomography scan shows a ground-glass nodule. **B** Illustration of measurement of the CTR. **C** The maximum diameter of the nodule is 22 mm. **D** The maximum diameter of the consolidation component is 11 mm

CT images were reconstructed using thin-section technology with a 1 mm collimation. Preoperative thin-section CT scans were examined with a lung setting using a window level of -600 Hounsfield units and a window width of 1800 Hounsfield units. Ground-glass nodules (GGN) were identified as lung nodules showing both ground-glass opacity (GGO) and consolidation components, while solid nodules were described as lesions without GGO components. Two independent observers assessed each lung nodule using the Picture Archiving and Communication Systems program. Two radiologists manually measured the longest diameter of each nodule and the maximum diameter of the consolidation component of each nodule using electronic calipers. CTR was calculated by dividing the maximum diameter of the consolidation component by the longest diameter of the nodule, as seen in the axial thin-section CT’s maximum section (Fig. 1).

### Pathologic evaluation

Tissues preserved in formalin and embedded in paraffin were cut into sections and then stained with hematoxylin and eosin, along with the Alcian blue-periodic acid-Schiff technique, to evaluate the production of cytoplasmic mucin. Two pathologists referenced the 5th edition of the World Health Organization classification to identify histologic typing and pathologic grade in tumors of the lung, pleura, thymus, and heart [12]. Atypical adenomatous hyperplasia (AAH) refers to a small, localized proliferation of mildly to moderately atypical type II pneumocytes and/or Clara cells along alveolar walls and sometimes respiratory bronchioles, typically measuring 0.5 cm or less; Adenocarcinoma in situ (AIS) is a small adenocarcinoma (≤ 3 cm) confined to neoplastic cells lining preexisting alveolar structures (lepidic growth) without invasion of stromal, vascular, or pleural tissues; Minimally invasive adenocarcinoma (MIA) is a solitary adenocarcinoma (≤ 3 cm) with a predominantly lepidic pattern and invasion of ≤ 5 mm in any one focus [13].

### Follow-up protocol

After surgery, patients were scheduled for follow-up appointments every three months for the first two years, then every six months from the third to the fifth year, and finally annually. These follow-up procedures consisted of regular chest and upper abdominal CT scans as well as head CT scans. The main goal of this research was to establish the overall survival and recurrence-free survival. OS was calculated from the date of surgery to the date of death due to any cause or the last follow-up. The RFS was determined starting from the first surgery and continuing until the first recurrence or the final clinical appointment.

### Statistical analysis

Continuous data is displayed as the average ± standard deviation (SD) or median (Q1, Q3) and were analyzed using either the Student’s t-test or the Mann-Whitney U test. Comparisons of categorical data were conducted using either Pearson’s chi-square test or the Kruskal-Wallis H test. X-title software was used to analyze the ideal threshold age of 56. Survival rates were compared among various groups by calculating survival curves with the Kaplan-Meier method and conducting the log-rank test. Important factors affecting survival in the initial analysis were incorporated into a multivariate Cox proportional hazard model. Cox models were utilized to assess the independent predictors of recurrence-free survival. Variables that had P values below 0.1 in the univariate analysis were included in a multivariate model. Statistical analyses were conducted using IBM Inc.‘s SPSS Statistics 26 and GraphPad Prism software. Statistical significance was attributed to a P-value of less than 0.05, and all reported significance levels were considered to be two-sided.

## Results

### Clinicopathologic comparison of surgical approaches

In Table 1, the characteristics of 564 surgically removed lung adenocarcinomas that are 2 cm or smaller are shown, categorized by type of surgery, with 266 lobar resections and 298 sublobar resections. Most patients in our study were female (61%), with a median age of 56. Tumors that underwent sublobar resection were typically 3 mm smaller compared to those that underwent lobar resection. AAH/AIS/MIA was more frequent in the sublobar-resected cohort than in the lobar-resected cohort (65% vs. 30%), whereas poorly differentiated tumors were less frequent (3.4% vs. 8.6%).

**Table 1:** Patient characteristics

Characteristics	Lobar resection (*N* = 266^1^)	Sublobar resection (*N* = 298^1^)	*p*-value^2^
**Sex**			0.21
Male	111 (42)	109 (37)	
Female	155 (58)	189 (63)	
**Age**	57 (49 – 64)	55 (48 – 64)	0.32
**Type**			<0.001
AAH/AIS/MIA	81 (30)	195 (65)	
IAC	185 (70)	103 (35)	
**Tumor size**	14.9 (11.1 – 17.5)	11.9 (9.5 – 15.5)	<0.001
**Solid size**	9.5 (4.7 – 14.2)	4.6 (0.0 – 7.9)	<0.001
**CTR**	0.72 (0.38 – 1.00)	0.40 (0.00 – 0.61)	<0.001
**Pathology grade**			<0.001
AAH/AIS/MIA/Well differentiated	107 (40)	212 (71)	
Moderately differentiated	136 (51)	76 (26)	
Poorly differentiated	23 (8.6)	10 (3.4)	

^1^n (%);Median(IQR). ^2^Pearson’s Chi-squared test; Wilcoxon rank sum test. AAH: atypical adenomatous hyperplasia; AIS: adenocarcinoma in situ; CTR: consolidation tumor ratio; IAC: invasive adenocarcinoma; MIA: minimally invasive adenocarcinoma

**Fig. 2:**
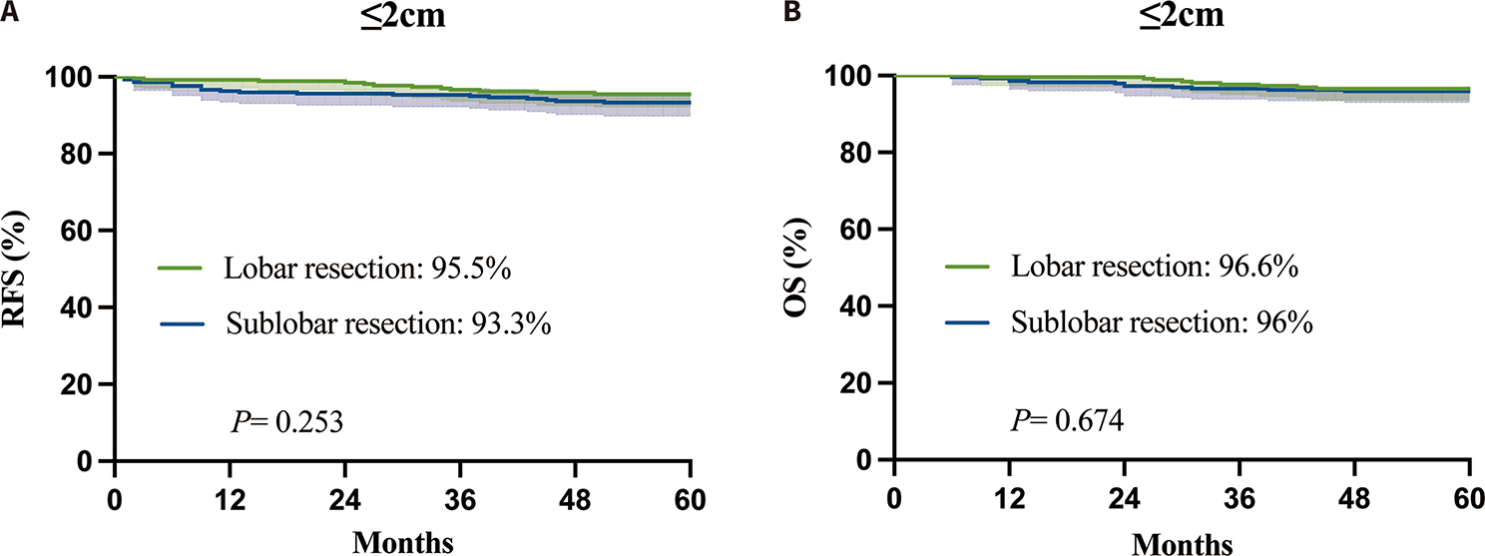
Survival outcome between lobar resection and sublobar resection groups in small(≤ 2 cm) early-stage lung adenocarcinoma(**A**, RFS; **B**, OS). The 5-year RFS and OS were similar between the lobar resection and sublobar resection groups. RFS, recurrence-free survival; OS, overall survival

Out of the 564 patients studied after surgery, 21 patients passed away (5 in the 0.7 ≤ CTR < 1 category and 16 in the CTR = 1 category), while 32 patients experienced recurrence (6 in the 0.7 ≤ CTR<1 category and 26 in the CTR = 1 category) over the observation period. The 5-year RFS and OS rates of all 564 patients were 94.3% and 96.3%, respectively. Figure 2 demonstrates that there were no disparities in mortality or recurrence rates between the sublobar resection and lobar resection groups over a 5-year period, with OS rates at 96% vs. 96.6% (*P* = 0.674) and RFS rates at 93.3% vs. 95.5% (*P* = 0.253). Table 2 shows the results of univariate and multivariate analyses for clinicopathological features associated with recurrence.

**Table 2:** Cox proportional hazard model for the 5-year recurrence-free survival in 564 patients

Variate	Univariate		Multivariate	
	HR (95%CI)	*P* value	HR (95%CI)	*P* value^1^
Gender(Female)	0.273(0.129, 0.576)	0.001	0.5(0.234, 1.066)	0.073
Age(>56 years)	6.171(2.377, 16.026)	<0.001	3.850(1.442, 10.273)	0.007
Maximum tumor size(D>1 cm)	2.773(0.973, 7.906)	0.056	1.130(0.186, 6.883)	0.895
Consolidation component size(D>1 cm)	11.382(4.684, 27.656)	<0.001	0.962(0.187, 4.938)	0.963
CTR(CTR≥0.75)	74.969(10.233, 549.218)	<0.001	82.952(8.732, 788.019)	<0.001
Surgical approach(Sublobar resection)	1.502(0.734, 3.073)	0.265	3.612(1.750, 7.453)	0.001

^1^P Value determined by COX proportional hazard model. CI: confidence interval; CTR: consolidation tumor ratio; D: diameter; HR: hazard ratio

In the univariate analysis of RFS, male sex, age > 56 years, Maximum tumor size > 1 cm, consolidation component size > 1 cm, and CTR ≥ 0.75 were associated with a lower RFS. Regarding the correlation between the CTR and pathology grade (*r* = 0.739, *P* < 0.001), we excluded the pathology grade from the multivariate analysis. Moreover, since the surgical approach has some impact on the prognosis of lung adenocarcinoma ≤ 2 cm, we enrolled the surgical approach in the multivariate analysis. Multivariate analysis identified that CTR ≥ 0.75(HR = 82.952, 95%CI 8.732, 788.019), age > 56 years (HR = 3.850, 95%CI 1.442, 10.273), and sublobar resection(HR = 3.612, 95%CI 1.750, 7.453) could predict worse survival.

### Survival outcomes according to CTR classification

**Fig. 3:**
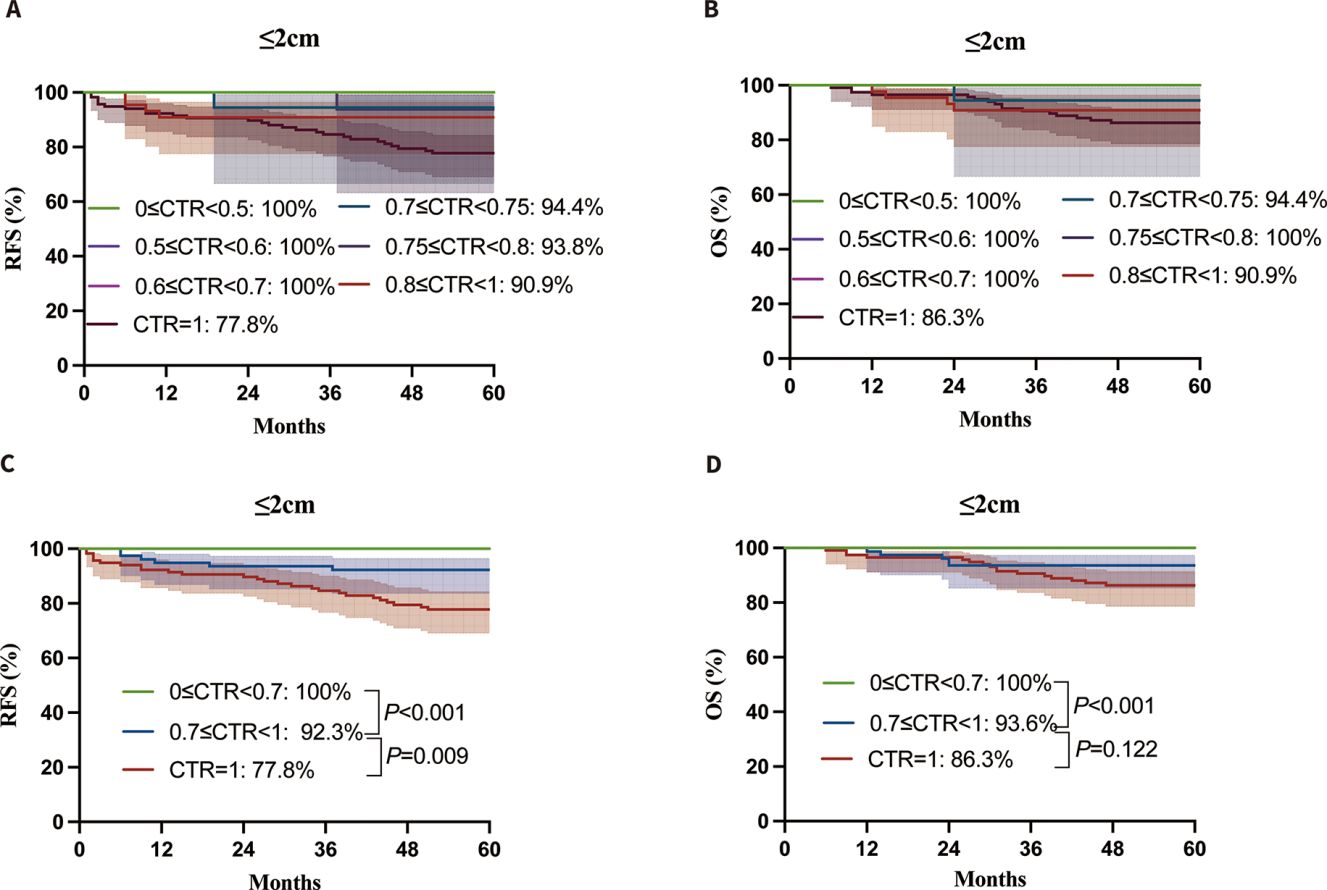
Survival outcomes according to CTR. There were no significant differences in RFS and OS among 0 ≤ CTR<0.5, 0.5 ≤ CTR<0.6, and 0.6 ≤ CTR<0.7 and in RFS and OS among 0.7 ≤ CTR<0.75, 0.75 ≤ CTR<0.8, and 0.8 ≤ CTR<1(*P* = 0.852 and *P* = 0.442, respectively); Significant differences existed in 5-year RFS and 5-year OS among 0 ≤ CTR<0.7, 0.7 ≤ CTR<1, and CTR = 1. CTR, consolidation-to-tumor ratio; RFS, recurrence-free survival; OS, overall survival

In the context of multivariate analysis, we delved deeper into the influence of CTR on survival by categorizing the 564 patients into six subgroups based on different ranges of CTR values: 0 ≤ CTR<0.5, 0.5 ≤ CTR<0.6, 0.6 ≤ CTR<0.7, 0.7 ≤ CTR<0.75, 0.75 ≤ CTR<0.8, 0.8 ≤ CTR<1, and CTR = 1. Figure 3A illustrates the 5-year RFS based on CTR, with rates of 100% for 0 ≤ CTR<0.5, 100% for 0.5 ≤ CTR<0.6, 100% for 0.6 ≤ CTR<0.7, 94.4% for 0.7 ≤ CTR<0.75, 93.8% for 0.75 ≤ CTR<0.8, 90.9% for 0.8 ≤ CTR<1, and 77.8% for CTR = 1. The 5-year overall survival rates based on CTR were as follows: 100% (0 ≤ CTR<0.5), 100% (0.5 ≤ CTR<0.6), 100% (0.6 ≤ CTR<0.7), 94.4% (0.7 ≤ CTR<0.75), 100% (0.75 ≤ CTR<0.8), 90.9% (0.8 ≤ CTR<1), 86.3% (CTR = 1) (Fig. 3B). It is evident that there were no notable variances in RFS and OS between the ranges of 0 ≤ CTR<0.5, 0.5 ≤ CTR<0.6, and 0.6 ≤ CTR<0.7. Additionally, there were no notable variances in RFS and OS between the ranges of 0.7 to less than 0.75, 0.75 to less than 0.8, and 0.8 to less than 1 (*P* = 0.852 and *P* = 0.442, respectively). Consequently, the patients were categorized into three groups based on their CTR values: CTR < 0.7, 0.7 ≤ CTR<1, and CTR = 1. The three groups showed notable variations in the 5-year RFS and 5-year OS, as depicted in Fig. 3C and D. As shown in Table 3, there were 369 CTR < 0.7, 78 0.7 ≤ CTR<1, and 117 CTR = 1. Patients with CTR > 0.7 comprised more males (*P* < 0.001) and more patients aged > 56 years (*P* < 0.001). In addition, patients in the CTR>0.7 groups had a higher solid component size (*P* < 0.001), a greater number of patients with low differentiation degree (*P* < 0.001), a higher number of patients diagnosed with IAC (*P* < 0.001), and a lower number of patients who underwent sublobar resection (*P* < 0.001) compared to the CTR<0.7 group.

**Table 3:** Clinicopathologic characteristics of 564 patients in CTR<0.7, 0.7≤CTR<1 and CTR=1 groups

Variable	CTR<0.7 (*N* = 369^1^)	0.7≤CTR<1 (*N* = 78^1^)	CTR=1 (*N* = 117^1^)	*p*-value^2^
**Sex**				<0.001
Male	122 (33%)	32 (41%)	66 (56%)	
Female	247 (67%)	46 (59%)	51 (44%)	
**Age**				<0.001
>56	159 (43%)	36 (46%)	75 (64%)	
≤56	210 (57%)	42 (54%)	42 (36%)	
**Type**				<0.001
AAH/AIS/MIA	265 (72%)	10 (13%)	1 (0.9%)	
IAC	104 (28%)	68 (87%)	116 (99%)	
**Tumor size**	12.0 (9.5, 15.6)	14.9 (12.4, 17.5)	16.2 (11.9, 18.4)	<0.001
**Solid size**	4.0 (0.0, 6.4)	12.1 (10.3, 14.2)	16.2 (11.9, 18.4)	<0.001
**Surgery approach**				<0.001
Lobar resection	130 (35%)	52 (67%)	84 (72%)	
Sublobar resection	239 (65%)	26 (33%)	33 (28%)	
**Pathology grade**				
AAH/AIS/MIA/Well differentiated	306 (83%)	12 (15%)	1 (0.9%)	
Moderately differentiated	61 (17%)	60 (77%)	91 (78%)	
Poorly differentiated	2 (0.5%)	6 (7.7%)	25 (21%)	

^1^n (%); Median (IQR). ^2^Pearson’s Chi-squared test; Kruskal-Wallis rank sum test; Fisher’s exact test. AAH: atypical adenomatous hyperplasia; AIS: adenocarcinoma in situ; CTR: consolidation tumor ratio; IAC: invasive adenocarcinoma; MIA: minimally invasive adenocarcinoma

### Comparison of survival rates in patients undergoing lobar resection versus sublobar resection with CTR values less than 0.7, between 0.7 and 1, and equal to 1

**Fig. 4:**
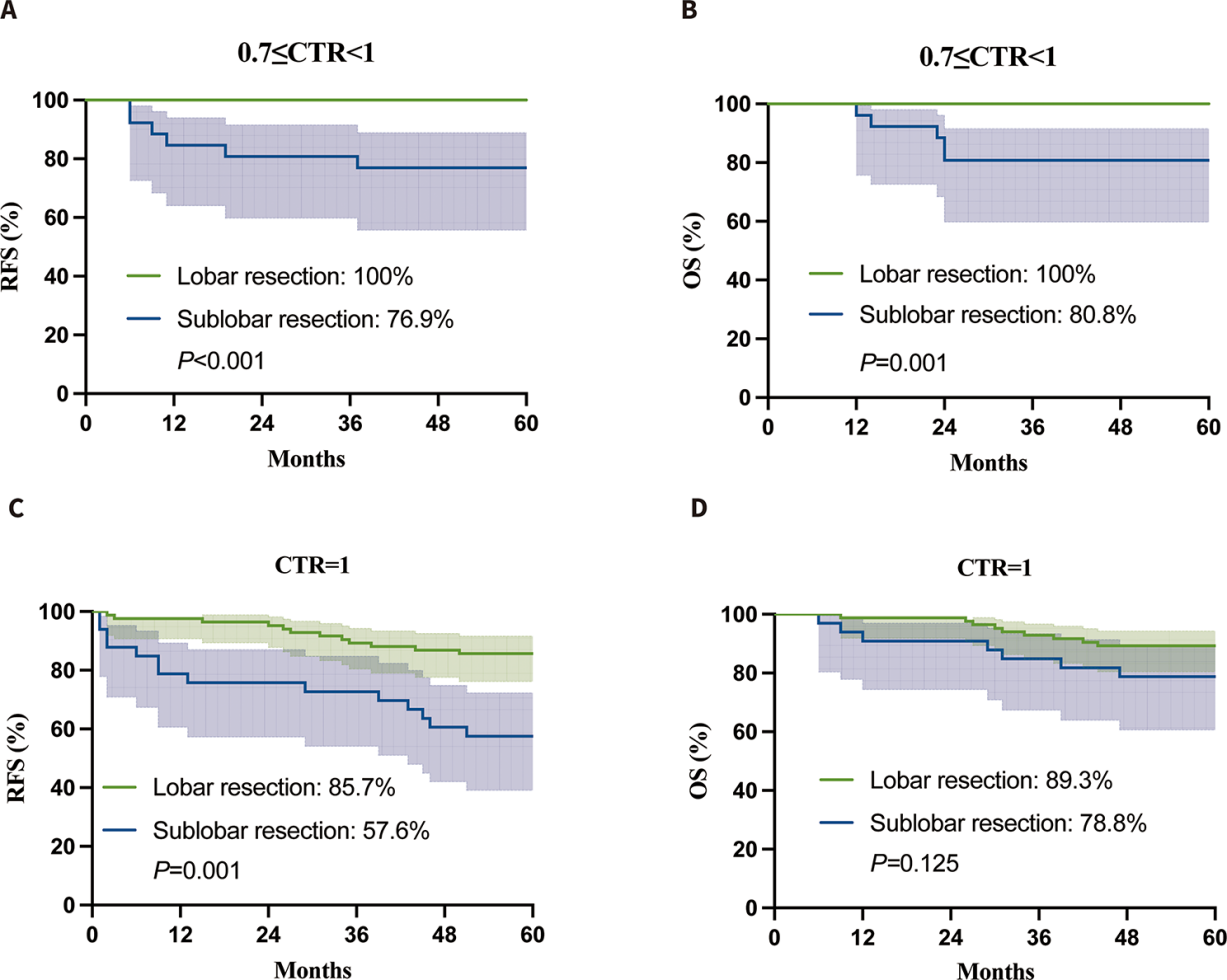
Survival outcomes between lobar resection and sublobar resection groups in 0.7 ≤ CTR<1(**A**, RFS; **B**, OS), and CTR = 1(**C**, RFS; **D**, OS). Both the 5-year RFS and OS were significantly different between the lobar resection and sublobar resection groups in 0.7 ≤ CTR<1(*P* < 0.001 and *P* = 0.001, respectively); AS for CTR = 1, the survival outcome was significantly different between the lobar resection and sublobar resection groups in RFS(*P* = 0.001). CTR, consolidation-to-tumor ratio; RFS, recurrence-free survival; OS, overall survival

After analyzing the results using the CTR classification, we found significant differences in both the 5-year RFS and OS between the lobar resection and sublobar resection groups with a CTR between 0.7 and 1. Specifically, the 5-year RFS was 100% vs. 76.9% (*P* < 0.001) and the 5-year OS was 100% vs. 80.8% (*P* = 0.001) as shown in Fig. 4A and B. In the case of CTR = 1, the 5-year recurrence-free survival rate was 85.7% for those who underwent lobar resection and 57.6% for those who had sublobar resection (*P* = 0.001) (Fig. 4C); the 5-year overall survival rate was 89.3% for lobar resection and 78.8% for sublobar resection (*P* = 0.125) (Fig. 4D). The data suggests that patients who underwent lobar resection had better clinical results compared to those who had sublobar resection, for both CTR values of 0.7 ≤ CTR<1 and CTR = 1. Yet, patients with CTR values between 0 and 0.7 showed no variance in RFS and OS outcomes when comparing lobar resection to sublobar resection groups (5-year RFS, 100% vs. 100%; 5-year OS, 100% vs. 100%). The sublobar resection group was shown to be just as effective as the lobar resection group when it came to CTR < 0.7.

### Survival outcomes among 0 ≤ CTR<0.7, 0.7 ≤ CTR<1and CTR = 1 in each surgery approach

**Fig. 5:**
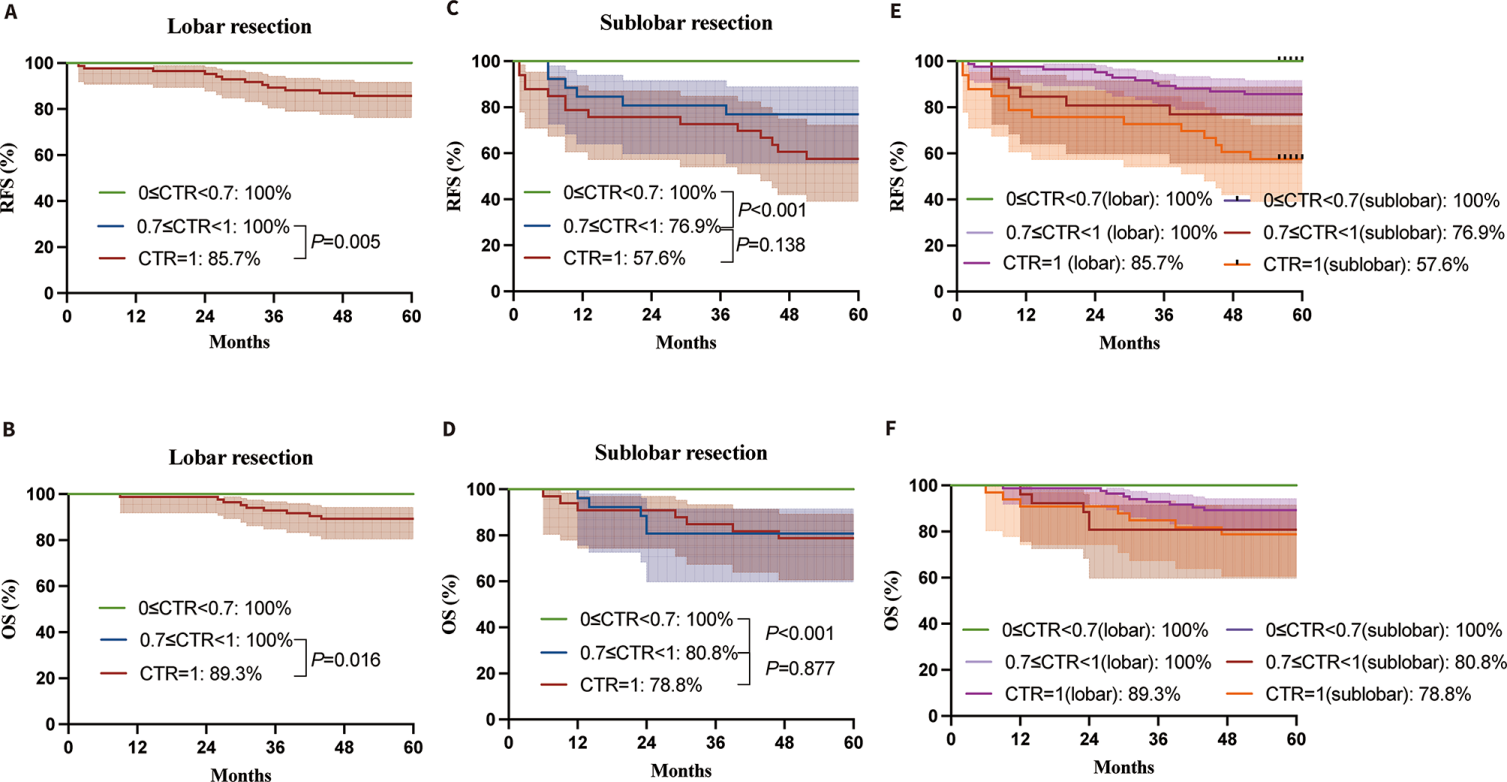
Survival outcomes among 0 ≤ CTR<0.7, 0.7 ≤ CTR<1 and CTR = 1 in lobar resection(**A**, RFS; **B**, OS) and sublobar resection(**C**, RFS; **D**, OS) and comparison of survival outcomes among them(**E**, RFS; **F**, OS). The 5-year OS of the lobar resection group was excellent, showing 89% or more regardless of CTR values. CTR, consolidation-to-tumor ratio; RFS, recurrence-free survival; OS, overall survival

The lobar resection groups showed similar RFS and OS between 0 ≤ CTR<0.7 and 0.7 ≤ CTR < 1, both at 100%, slightly higher than CTR = 1 (*P* = 0.005 and *P* = 0.016), as depicted in Fig. 5A and B. In the sublobar resection groups, however, 0 ≤ CTR<0.7 had a significantly higher RFS (Fig. 5C) and OS (Fig. 5D) compared with 0.7 ≤ CTR < 1 (*P* < 0.001 and *P* < 0.001). The 5-year RFS and OS of 0 ≤ CTR<0.7 with sublobar resection was equivalent to that of 0 ≤ CTR<0.7 with lobar resection.

A CTR between 0.7 and 1 after lobar resection showed similar 5-year RFS and OS rates compared to a CTR between 0 and 0.7 after sublobar resection (Figs. 5E and F and 100% vs. 100%). Nevertheless, a CTR of 1 following lobar resection resulted in notably reduced RFS and OS in comparison to a CTR between 0.7 and 1 following lobar resection (*P* = 0.005 and *P* = 0.016) (Fig. 5E and F). A CTR of 1 after lobar resection showed similar 5-year RFS and OS compared to a CTR of 0.7 to less than 1 after sublobar resection (*P* = 0.243 and *P* = 0.203, respectively) (Fig. 5E and F).

## Discussion

On the basis of published results of CALGB140503 [14] and JCOG0802 [15] demonstrating favorable survival time of sublobar resection, compared to lobar resection, for peripheral IA1 NSCLC with consolidation-to-tumor ratio(CTR)>0.5. this study aimed to evaluate the clinical utility values of the CTR in the management of patients with small(≤ 2 cm) early-stage lung adenocarcinoma. The strong points of this study are that the sample size is sufficient and it strongly demonstrates that there is no difference in RFS and OS between lobar and sublobar resection, until the CTR > 0.7, which is significant. That is individuals with tumors having a CTR ≥ 0.7 who undergo sublobar resection face a greater likelihood of recurrence compared to those who undergo lobar resection. Besides, our current research shows that sublobar resection was as effective as lobar resection for patients with a CTR < 0.7.

Prior research has shown various findings regarding the outlook of PSNs and solid nodules in lung adenocarcinomas with a total tumor size of 2 cm or less. Two large randomized surgical control trials (JCOG0802/WJOG4607L and CALGB140503)enrolling 1106 and 697patients, respectively, have shown noninferiority of sublobar resection compared to lobar resection for peripheral NSCLC radiographically measuring ≤ 2 cm [14, 15]. Research from Germany [16] also reported that overall survival and locoregional and distant recurrences were not significantly different for patients undergoing either sublobar resection or lobar resection for stage IA adenocarcinoma. Our results were in line with the above studies in that the survival outcome of sublobar resection was similar to that of the lobar resection group. The prognostic equivalence between lobar and sublobar resection was presumably because all nodules were analyzed together, regardless of the prognostic indicator. Ma et al. [17] demonstrated that vascular invasion is an independent prognostic factor for lung adenocarcinomas ≤ 2 cm. In their research, vascular invasion could predict the subgroup of lung adenocarcinoma ≤ 2 cm at risk of poor outcome treated by wedge resection compared to lobectomy. Furthermore, data from the National Cancer Database [10] showed that lobar resection was superior to sublobar resection for high-grade NSCLC (≤ 2 cm). In our study, CTR ≥ 0.75, age > 56 years, and sublobar resection could predict worse survival.

Despite previous concerns, the latest research uncovered important prognostic variances between lobar excision and sublobar excision of pulmonary nodules with a total tumor size under 2 cm according to the CTR. In this study, our results were in line with those of previous studies showing that sublobar resection was not inferior to lobar resection in patients with CTR < 0.7 [18]. However, among patients with CTR ≥ 0.7, sublobar resection was associated with poorer outcomes than lobar resection, which is consistent with previous studies [19, 20]. In contrast, our results were inconsistent with those of Li et al. [21], who showed that sublobar resection could achieve oncological outcomes comparable to lobar resection for pure solid small-sized NSCLC. This contradiction may be caused by our cases precluding lymph node metastasis, while the lobar resection group had a higher rate of lymph node metastasis than the sublobar resection arm, acquiring more positive events in the lobar resection group. Wu et al. [22] reported that sublobar resection could achieve superior perioperative outcomes and equivalent oncological efficacy compared with lobar resection in elderly patients (≥ 75 years old) with peripheral solid-dominant and diameter ≤ 2 cm NSCLC. This difference may be due to the differences in the proportion of poorly differentiated cases. It is well known that the worse the differentiation, the worse is the prognosis [23]. In our study, CTR correlated well with the pathology grade (*r* = 0.739, *P* < 0.001); as the CTR increased, the percentage of moderate/poorly differentiated patterns increased and was highest in the CTR = 1 group. Our results are supported by those of other researchers in Korea. Yoon et al. [24] revealed that CTR reflects the predominant patterns of invasive components, suggesting that CTR as a useful imaging biomarker should be considered in the management of early-stage lung cancer.

A noteworthy discovery in the recent research was that patients who underwent lobar resection had lower RFS and OS when their tumors had a CTR = 1, as opposed to tumors with a CTR between 0.7 and 1. Tumors with CTR = 1 had more pleural, lymphatic, or vascular invasion and pathological lower grade than tumors with CTR < 1 [5, 24]. Wang et al. [25] reported that adjuvant chemotherapy was a favorable prognostic factor for micropapillary-predominant lung adenocarcinoma in stage IA. Naziye et al. [26] showed that adjuvant platin-based chemotherapy had a longer 5-year RFS in patients with small tumors with adverse risk factors such as visceral pleural effusion, lymphovascular invasion, grade 3, and solid micropapillary pattern. Thus, lobar resection may not be the sole treatment for lung ADC with a CTR of 1, and adjuvant chemotherapy might be added.

This study had some limitations. Additional clinicopathological characteristics were examined in initial studies, however, their associations with the likelihood of recurrence or death were uncertain and are not included in this report. Furthermore, manual measurement of CTR values may introduce measurement inaccuracies. In the future, we anticipate utilizing more precise measurement techniques, including artificial intelligence, to improve the accuracy of CTR value measurements.

### In conclusion

Lobar resection is associated with better long-term survival outcomes than sublobar resection for small lung adenocarcinomas ≤ 2 cm and CTR ≥ 0.7.

## Data Availability

Please contact the corresponding author with a request for data.

## Abbreviations

AAH: Atypical adenomatous hyperplasia
AIS: Adenocarcinoma in situ
CI: Confidence interval
CT: Computed tomography
CTR: Consolidation-to-tumor ratio
GGNs: Ground-glass nodules
GGO: Ground-glass opacity
IAC: Invasive adenocarcinoma
IASLC: International association for the study of lung cancer
JCOG: The Japan clinical oncology group
MIA: Minimally invasive adenocarcinoma
NSCLC: Non-small cell lung cancer
OS: Overall survival
RFS: Recurrence-free survival
SNs: Solid nodules
